# Single Intense Microsecond Electric Pulse Induces Radiosensitization to Ionizing Radiation: Effects of Time Intervals Between Electric Pulse and Ionizing Irradiation

**DOI:** 10.3389/fonc.2018.00418

**Published:** 2018-09-27

**Authors:** Zohre Rezaee, Ali Yadollahpour, Vahid Bayati

**Affiliations:** ^1^Department of Medical Physics, School of Medicine, Ahvaz Jundishapur University of Medical Sciences, Ahvaz, Iran; ^2^Cellular and Molecular Research Center, Ahvaz Jundishapur University of Medical Sciences, Ahvaz, Iran

**Keywords:** electroporation, ionizing radiation, radiosensitizing effect, time interval, dose enhancement factor

## Abstract

**Background and Objective:** Recent studies have shown the potential of electroporation (EP) as a physical radiosensitizer for ionizing radiation (IR). The amount of sensitizing effect depends on some factors the most important of them is the time interval between the EP and IR. This experimental *in vitro* study aims to investigate the radiosensitizing effect of EP exposure prior to IR and also evaluate the effects of EP-IR time intervals on the amount of radiosensitizing effects.

**Methods:** Chinese hamster ovary (CHO) cell lines were cultured *in vitro*. The cells were divided into 10 groups including one untreated or control group, IR, and EP treatment alone groups, and seven combined EP-IR groups with 10, 20, 30, 40, 50, 60, and 70 min intervals. The dose enhancement factors (DEFs) for 6 MV X-rays IR were comparatively investigated between the groups using MTT assay.

**Results:** The EP significantly induced radiosensitizing effect and its amount depends on the time intervals. The viability rate of the cells in the combined EP-IR treatment groups for intervals of 10, 20, 30, 40, and 50 min was significantly lower than the IR alone group. The highest DEF (1.18) was observed 10 min time interval between EP and IR.

**Conclusion:** The radiosensitizing effects of EP persist long enough, 10–50 min, which allows safe application of EP as a radiosensitizer before IR in clinical setting.

## Introduction

Cancer is the leading cause of human mortality worldwide (1). Radiation therapy (RT) is an important modality of cancer treatment (2, 3). In this method, the growth of tumor cells is controlled by bombardment with ionizing radiation, causing DNA damage by direct action or through formation of free radicals by indirect action (4, 5). However, RT can also induce unwanted damages to the normal surrounding tissues due to little discrimination between malignant and normal cells. In addition, the locoregional tumor progression following RT increases the mortality rates of this technique. Therefore, developing new improvements addressing these issues is necessary. Developing targeted drug delivery techniques along with efficient radiosensitizing agents can efficiently address these issues through enhancing targeted uptake of the anticancer agents into tumor cells and selective sensitizing tumor cells to ionizing radiation (IR) (6, 7). Electroporation (EP) is one of these modalities with promising outcomes as both targeted drug delivery system and radiosensitizing technique (8). EP increases uptake of antitumor agents in the tumor cells and their intracellular accumulation which result in radiosensitizing effects (9, 10). EP applies a single or repeated high voltage, short-duration electric pulse over the target cells inducing transient pores across membrane which in turn significantly increases the cell membrane permeability to ions and macromolecules (11–14). The induced pores are created rapidly approximately within 10^−6^ s and disappear within few seconds to several minutes after exposure to the electric field (12, 13).

EP is a highly effective technique to increase the cell membrane permeability by application of high voltage, short-duration electric pulses (9, 10). Indeed, when the cell membrane is exposed to an external electric field, a transmembrane potential is induced (12, 13, 15, 16). If the induced transmembrane potential is sufficiently high, dielectric breakdown leading to generate a phase transition that causes nano or micro-pores to form in lipid bilayers membrane (17, 18). During this step, the conductivity of membrane is increased rapidly (19). Therefore, the intracellular electric field increases, which may induce biochemical changes inside of the cell (20).

Some studies have shown EP, alone or in combination with other modalities, can induce radiosensitizing effects in different cancerous cells which consequently reduce the required total absorbed dose in radiotherapy (6, 7, 21).

Gabriel et al have reported that when the cells are exposed to electric pulses, the reactive oxygen species (ROS) is generated in the electropermeabilized part of the cell membrane. ROS can sensitize the cells to ionization radiation (22). Therefore, the radiation dose in the site of tumor can be selectively amplified by using EP prior to irradiation. Different studies have shown the radiosensitizing effects of EP, however, it is not clear how long do the effects persist. In this study we try to answer this question. The present study aims to investigate the effect of time intervals between EP and IR on the amount of radiosensitizing effects *in vitro*. In addition, in context of this study, we try to discuss the findings of our recent studies as well as current literature on the radiosensitizing effects and probable mechanisms of EP alone and in combination with other modalities in different cell lines. To the best of our knowledge this is the first time that the effects of time interval in different intervals (10–70 min) between EP and IR has been shown.

## Methods

### Cell culture

Chinese hamster ovary (CHO) cell lines were purchased from National Cell Bank of Pasteur Institute of Iran (NCBI, C111). The cells were grown in the Roswell park memorial institute (RPMI) 1640 medium (Bio-Idea, Iran) supplemented with 10% Fetal bovine serum (FBS; Gibco), %1 penicillin/streptomycin (Bio-Idea, Iran). Cells were routinely subcultured twice a week using 0.25% trypsin-EDTA (Bio-Idea, Iran) and were maintained in T-75 flasks in CO_2_ incubator (RS Biotech Galaxy R) under standard condition (37°C, 5% CO_2_).

### Electroporation protocol

The cells from the exponential growth phase were harvested from monolayer cultures using trypsin-EDTA. Following the trypsination stage, the cells were centrifuged for 5 min at 1,500 rpm (Centrifuge Hettich, Protofix 32 A) and resuspended in RPMI 1640 medium at a concentration of 1 × 10^6^ cells/ml. Finally, 30 μl cell suspension was added into Bio-Rad cuvettes with a 1-mm gap. The sample was exposed to one square electric pulse with electric field intensity 1200 V/cm and pulse duration 100 μs. EP of CHO cell line was carried out using a Bio-Rad Gene Pulser Xcell™ Electroporation system. After completion of delivering pulse, the sample was transferred to 96-well plate and the fresh medium was added to cells. In EP alone group, the 96-well plate was incubated for 24 h and then harvested for analysis. In contrast, in the EP+ IR groups, the 96-well plate was irradiated after different time intervals and then incubated for 24 h.

### Ionizing radiation of sample

The IR treatments were carried out using 6 MV X-rays through Varian 2100 C/D linear accelerator (LINAC, Golestan Hospital, Ahvaz, Iran). The samples received a total dose of 2 Gy with a field size of 10 × 10 cm^2^ at source-to-surface distance (SSD) of 100 cm. To produce appropriate build up for the 6 MV beam, a Plexiglass sheet (water equivalent) with 1.5 cm thickness was placed on the top of the 96-well plate. In addition, to reach a sufficient generation of backscatter, 3 cm thickness of a Plexiglass sheet was utilized under the bottom of 96-well plate.

### Experimental groups

To determine whether EP can sensitize the cells to mega voltage IR and to obtain the optimal time interval between EP and irradiation, 10 experimental groups were designed in this study: untreated tumors, tumors treated with IR or EP alone, and seven groups with time intervals irradiated after 10, 20, 30, 40, 50, 60, and 70 min after EP (EP + IR). All of the measurements in the experimental groups were performed at least three times to increase the reliability and accuracy of the results and mean values were used for further analyses. The repetitions were biologically in which the experiments were performed in different dates from different cell cultures and resulting cell suspensions and the values were averaged.

### MTT assay and determination of cell viability

The viability rate of cells in different groups as an indicator of cell response to irradiation was evaluated by MTT assay kit (Bio-Idea, Iran). In this colorimetric method, the mitochondrial dehydrogenase activity of proliferating cells reduce the MTT salt (3-[4,5-dimethylthiazol-2-yl]-2,5-diphenyltetrazolium bromide) to purple MTT formazan crystals. Therefore, upon the completion of treatments, cells were incubated 24 h at 37°C with 5% CO_2_ in atmosphere. Twenty four hours later, according to manufacturer's instructions, the culture medium was removed and 10 μl MTT solution (5 mg/ml) and 100 μl ready to use RPMI1640 culture media (without phenol red) were added to each well and plate was incubated for an additional 4 h. Then MTT was replaced by 50 μl dimethyl sulfoxide (DMSO) solution and the plate was shaken for 10 min by orbital shaker. The optical density (OD) of each well was assessed using spectrophotometer (Model 680, BIO-RAD) at a 570-nm test wavelength. The experiments were performed in triplicate, thus we used the average OD to obtain cell viability rate. This factor for each group was calculated the following formula: viability rate = (average OD_570nm_ of treated group*/*average OD_570nm_ of the untreated group) × 100%. In addition, the dose enhancement factor (DEF) was obtained by dividing the viability rate of EP + IR group to IR group.

### Statistical analysis

The normality of data was assessed by Kolmogorov-Smirnov test. The statistical differences between experimental groups were carried out by one-way ANOVA. If differences were significant, the *post hoc* test was applied to compare groups. All statistical analyses were performed with SigmaStat statistical software (Systat Software, Inc) and *p*-values ≤ 0.05 were considered statistically significant. The values are represented as Mean ± standard error of mean (SEM).

## Results

**Figure 1:**
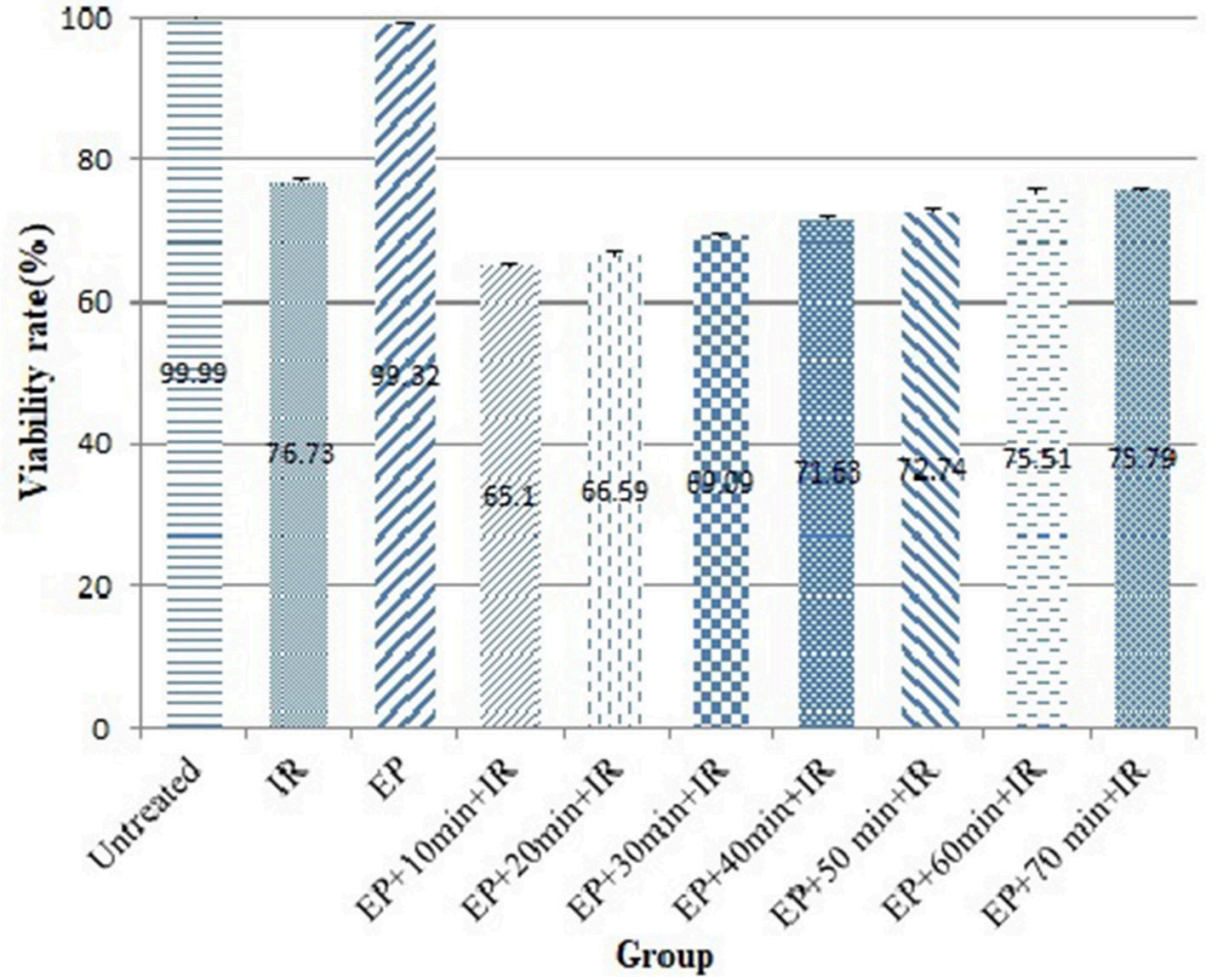
Viability rates of cells after treatment by different protocols.

The effect of time interval between EP and IR on radiosensitizing effect of EP was investigated in CHO cell lines. As shown in Figure 1 and Table 1, the viability rate decreased from 99.99% in untreated group to 76.73% in the cells treated with IR alone. When the cells were exposed to electric pulse alone, the viability rate reached 99.32% and had no significant difference with the untreated group (*P* = 0.976). However, combination of EP with IR increased the response of cells to treatment which was statistically significant. When EP was performed 10 min prior to irradiation, the lowest viability (65.10%) was observed (*P* = 0.0001).

**Table 1:** Comparison of viability rates and dose enhancement factors of the CHO cells between the different experimental groups.

**Group**	**Viability rate (%)**	**DEF**
Untreated	99.99 ± 0.36	–
IR	76.73 ± 0.43	–
EP	99.32 ± 0.23	–
EP+10 min + IR	65.10 ± 0.26	1.18
EP+20 min + IR	66.59 ± 0.58	1.15
EP+30 min + IR	69.09 ± 0.54	1.11
EP+40 min + IR	71.63 ± 0.44	1.07
EP+50 min + IR	72.74 ± 0.28	1.05
EP+60 min + IR	75.51 ± 0.43	1.016
EP+70 min + IR	75.79 ± 0.50	1.012

*The values are represented as Mean ± standard error of mean (SEM). All of the experiments were performed three times and the averaged values are calculated for further analyses. IR, Ionizing radiation; EP, Electroporation*.

The dose enhancement factor (DEF) in this group was 1.18. By exposing of electric pulse 20 min before to irradiation, the survival rate, and DEF reached to 66.59% and 1.15, respectively. There was no significant difference between time interval of 10 and 20 min in the viability rate and DEF (*P* = 0.340). When the time interval was 30 min, the viability rate and DEF of 69.09% and 1.11 were achieved. By increasing the time interval from 40 to 50 min, survival was increased from 71.63 to 72.74% and DEF was decreased from 1.07 to 1.05. Treatment of cells with EP 60 min and 70 min prior to IR resulted in 75.51 and 75.79% viability rate, respectively that was similar to viability rate in the group received irradiation alone (*P* = 0.593 and *P* = 0.846, respectively). It seems, after 50 min of delivering electric pulse, the radiosensitizing effects induced by EP decreased to insignificant levels. Therefore, to obtain the highest cell response, it is essential to deliver electric pulse 10–50 min before irradiation.

## Discussion

This study investigated the radiosensitizing effects of a single 100 μs pulse of EP to 6 MV X-rays IR in CHO cell lines *in vitro*. Moreover, the effects of time intervals between EP and IR on the amount of radiosensitizing effect were studied. Our findings showed that EP significantly increased the response rate in the CHO cells *in vitro* in a time dependent manner where the highest amount of radiosensitizing effect with DEF of 1.18 was observed for 10 min EP-IR interval that was significantly higher than IR alone group. This difference in the viability rate of the cells between the combined EP-IR and IR alone group remained significant for intervals of 20, 30, 40, and 50 min and then decreased. Our findings showed EP can be used 10 to 50 min before IR to induce significant radiosensitizing effects.

In normal physiological conditions, the electric conductivity of cytoplasm and extracellular medium is greater than the conductivity of the cell membrane. Therefore, under the exposure of an external electric field to the lipid membrane, the anode- and cathode-facing side, respectively becomes hyper-polarized and depolarized and a transmembrane potential is induced on the exposed cell (19, 23, 24). Reversible EP is the biophysical process that transiently increases the permeability of cell membrane through the application of short, intense electric pulses (25–27). EP is routinely employed to transport nonpermeant molecules such as DNA, dyes, proteins, and chemotherapeutic agents into the cell (28–30). This technique can modulate the intrinsic functional characterization of the target cell and increase the oxidative burst within the cell medium. Previously, this phenomenon has been observed in neutrophil (31), macrophage (32), and lymphocyte (33) cells. Gabriel et al reported that EP can induce oxidative jump and generate reactive-oxygen species (ROS) in CHO cells (22). The electro-induced oxidative jump can be appeared when the applied electric field intensity is higher than critical threshold value that is controlled by duration of pulse. To simultaneously trigger the electropermeabilization and electro-induced ROS production, this critical value is 0.44 V/cm (34). The electro-induced ROS generation is not homogenous and restricted to the electropermeabilized part of the cell membrane (35). Therefore, this technique has a potential to be combined by IR to increase lethal damage of irradiation. The efficiency of this combined modality (electro-radiotherapy) is determined by quality of radiation, electric pulse parameters, and time interval between EP and radiotherapy. In this present study, we focused on the effect of time interval between EP and radiotherapy. Our data are in agreement with previous studies investigating the radiosensitizing effects of EP alone or in combination with other modalities. West et al. (36) were probably the first group to report the interaction between EP and lethal damages of radiation. They investigated the effects of EP on the viability rate of the CHO cells *in vitro* at three time intervals between EP and Cs^137^- γ-radiation: immediately, 1 and 24 h. They reported that application of one exponential decaying electric pulse immediately prior to irradiation increased both α and β parameters of survival curve and the DEF to 1.19. However, EP applied 1 or 24 h before irradiation had no sensitizing effect on CHO cell line. Our study confirmed these results and also revealed that the optimal time interval to deliver electric pulse prior to radiotherapy is 10–50 min and applying electric pulse 60–70 min before radiotherapy has no effect on output of radiation treatment. Moreover, in our study EP could enhance the effect of radiation by factor of 1.18 (for time interval of 10 min). The findings of the Sersa group (21) revealed that EP can improve the effect of 220 KV radiation in tumor-bearing animals. The main purpose of their work was to investigate whether EP as a drug delivery system can increase the radiosensitizing effect of cisplatin as a chemotherapeutic drug, but they also observed that delivering of electric pulses prior to irradiation even in the absence of cisplatin, could enhance the response of cell to radiation. Kranjc et al reported the similar results where irradiation of LPB sarcoma cells that pretreated by electric pulses (without drug) enhanced cytotoxicity of radiation (EF = 1.25) (37).

The radiosensitizing effects of EP are not limited to CHO cell lines. Some studies have reported the radiosensitizing effects of EP in HT-29 cells which are highly radioresistant (7). In other *in vitro* study conducted by our group, we investigated the radiosensitizing effects EP, gold nanoparticles (GNPs) alone and combined EP-GNPs in the HT-29 and CHO cell lines in different conditions (7). Our results showed that EP could sensitize both HT-29 and CHO cell lines to 6 MV X-ray with a DEF of 1.36 and 1.28, respectively (1). The findings showed that EP and GNPs alone, and combined EP-GNPs significantly enhanced the response of cells to irradiation. Moreover, combined EP-GNPs showed synergistic radiosensitizing effect. However, the synergistic effect was observed just for HT-29 tumor cell lines. In other study by our group, we observed that the survival fraction of the HT-29 cells was significantly decreased by EP prior to radiotherapy. A single electric pulse of 100 μs increased the sensitivity of colorectal HT-29 cancer cell to megavoltage radiation by a factor of 1.36. The LD50 was decreased from 3.97 Gy in radiation alone group to 2.9 Gy in tumors treated with EP before irradiation which resulted in the sensitizer enhancement ratio of 1.36 (38).

Radiosensitizing effects of EP have been reported for different IR energies and also different types of the radiations ranging orthovoltage KV to MV X-rays. In this regard, West et al. have reported the radiosensitizing effects of EP to γ-radiation of the radioisotope sources of Cs^137^ (36) and Shil et al. to the γ-radiation of the radioisotope of Co^60^ (8). Moreover, the radiosensitizing effects of EP in lower energies of orthovoltage X-rays of 100-500 KV have been reported (21, 39, 40). However, nowadays, radioisotope sources and orthovoltage unit are gradually replaced by linear accelerators thus we used a LINAC as a radiation source to generate mega-voltage X-ray. That can be used to treatment of deep tumors.

Regarding the mechanisms of actions of radiosensitizing effects of EP, different studies have been conducted. Generation of oxidative jump at the electroporated sites of membrane and production of ROS is the probable mechanism of EP radiosensitization (34, 35). The level of generated ROS after EP was measured by Shil et al. in which they reported that the ROS level under the combined EP-irradiation group was significantly higher than irradiation alone group (8). Shil et al demonstrated that EP can significantly increase the generated ROS level in the Ehrlich Ascites Carcinoma (EAC) cells. In addition, for *in vivo* experiments, on the 7th day after treatment, the average tumor volume of electro-radiotherapy group was significantly (51%) smaller than this volume in control group (8).

## Conclusion

In conclusion, the results of present study demonstrate that the best time of EP to induce radiosensitizing effect to megavoltage irradiation, is 10 min before IR. This radiosensitizing effect is lost after 60 min. In view of clinical applications, the 10–50 min is long enough to apply EP as a radiosensitizing approach to IR to increase the response rate in cancer treatment. However, further studies in different cell lines and also *in vivo* conditions are needed to reach a definitive conclusion and using the EP in clinical setting.

## Author contributions

ZR, AY, and VB contributed equally in notion, study design and implementing, and data collection and manuscript preparation.

### Conflict of interest statement

The authors declare that the research was conducted in the absence of any commercial or financial relationships that could be construed as a potential conflict of interest.
